# Stoma versus anastomosis after sphincter-sparing rectal cancer resection; the impact on health-related quality of life

**DOI:** 10.1007/s00384-022-04257-w

**Published:** 2022-09-26

**Authors:** Jelle P. A. Algie, Robert T. van Kooten, Rob A. E. M. Tollenaar, Michel W. J. M. Wouters, Koen C. M. J. Peeters, Jan Willem T. Dekker

**Affiliations:** 1 Department of Surgery, Leiden University Medical Center, Albinusdreef 2, J10-71, 2333 ZA Leiden, The Netherlands; 2 Department of Biomedical Data Sciences, Leiden University Medical Center, Leiden, The Netherlands; 3 Department of Surgery, Netherlands Cancer Institute-Antoni Van Leeuwenhoek, Amsterdam, The Netherlands; 4 Department of Surgery, Reinier de Graaf Gasthuis, Delft, The Netherlands

**Keywords:** Rectal cancer, Stoma, Surgery, Health-related quality of life

## Abstract

**Background:**

Surgical resection is the mainstay of curative treatment for rectal cancer. Post-operative complications, low anterior resection syndrome (LARS), and the presence of a stoma may influence the quality of life after surgery. This study aimed to gain more insights into the long-term trade-off between stoma and anastomosis.

**Methods:**

All patients who underwent sphincter-sparing surgical resection for rectal cancer in the Leiden University Medical Center and the Reinier de Graaf Gasthuis between January 2012 and January 2016 were included. Patients received the following questionnaires: EORTC-QLQ-CR29, EORTC-QLQ-C30, EQ-5D-5L, and the LARS score. A comparison was made between patients with a stoma and without a stoma after follow-up.

**Results:**

Some 210 patients were included of which 149 returned the questionnaires (70.9%), after a mean follow-up of 3.69 years. Overall quality of life was not significantly different in patients with and without stoma after follow-up using the EORTC-QLQ-C30 (*p* = 0.15) or EQ-5D-5L (*p* = 0.28). However, after multivariate analysis, a significant difference was found for the presence of a stoma on global health status (*p* = 0.01) and physical functioning (*p* < 0.01). Additionally, there was no difference detected in the quality of life between patients with major LARS or a stoma.

**Conclusion:**

This study shows that after correction for possible confounders, a stoma is associated with lower global health status and physical functioning. However, no differences were found in health-related quality of life between patients with major LARS and patients with a stoma. This suggests that the choice between stoma and anastomosis is mainly preferential and that shared decision-making is required.

**Supplementary Information:**

The online version contains supplementary material available at 10.1007/s00384-022-04257-w.

## Introduction

With an estimated 704,000 new patients worldwide each year, rectal cancer has become the eighth most diagnosed cancer type in the world in 2018 [1]. Approximately 3,300 new patients are diagnosed with rectal cancer in the Netherlands every year [2]. Of these patients, 63.6% receive a (temporary) stoma [3]. Nowadays, the treatment of rectal cancer is adopting a more multimodal approach, but surgical resection is still the cornerstone of curative treatment [4]. Over the past decades, the 5-year survival has gone up to 75–80% [5]. The increased survival over the past decades and enlarged focus on value-based healthcare account for the growing interest in the quality of life after cancer treatment [6–8]. An example is the shift from abdominoperineal excision (APE) to sphincter-sparing techniques with low anastomosis in order to maintain organ preservation and bowel continence [9]. The ongoing upswing in overall survival after rectal cancer surgery brings about new dilemmas such as stoma presence, bowel dysfunction, and psychological and physical stress [10, 11].

After rectal cancer resection, surgeons are left with the decision on how to reconstruct. Should an anastomosis be constructed with or without a defunctioning stoma or should a definitive stoma be made? For this choice, two considerations are key: first of all, the risk of anastomotic leakage, its consequences, and whether a patient is able to cope with them [12]. An anastomotic leak can be a fatal insult to a frail patient. The other important consideration is the risk of a poor functional outcome. Approximately 41% of patients without a stoma after a sphincter-sparing surgical resection for rectal cancer experience major low anterior resection syndrome (LARS) 1 year after surgery [13]. LARS is described as a “disorder of bowel function after rectal resection, leading to a detriment in quality of life” [14, 15]. Frequently (≥ 35%) reported symptoms are clustering of bowel movement, incomplete evacuation, fecal incontinence, uncontrollable flatus, and urgency [16]. LARS has been shown to have a detrimental influence on short- and long-term health-related quality of life [17, 18]. Factors that have a negative impact on functional outcomes after rectal resection are low anastomosis, temporary stoma, or a stoma before surgery and (neo-)adjuvant radiotherapy. A definitive stoma may prevent these adverse functional outcomes. However, also, stoma-related complications such as parastomal hernia, retraction, prolapse, and stoma necrosis must be considered [19, 20]. This also goes for temporary stoma’s as they can significantly increase mid- to long-term morbidity and cause readmissions and re-interventions. Furthermore, up to 28.5% of temporary stomas are never reversed [21].

Post-operative complications, poor functional outcomes, and the presence of a stoma in patients may all influence the quality of life after surgery, making the decision between the formation of a (temporary) stoma or anastomosis a difficult one [22]. This decision should always be made together with the patient. Information on quality of life after rectal cancer surgery is vital for shared decision-making [23]. This study aims to determine the influence of a stoma on the health-related quality of life (HRQoL) after rectal cancer surgery and gain more insights into the trade-offs between stoma and anastomosis on the long run. In addition, the difference in HRQoL between patients with major LARS and a stoma is analyzed, using patient-reported outcome measures (PROMs).

## Methods

### Study population and treatment

The Medical Ethics Committee Leiden Den Haag Delft assessed this study protocol and concluded no formal review was needed, as this study is not being conducted under the Medical Research Involving Human Subjects Act (WMO). Consecutive patients who underwent surgical resection for rectal cancer in the Leiden University Medical Center, Leiden, The Netherlands, and the Reinier de Graaf Gasthuis, Delft, The Netherlands, between January 2012 and January 2016 with at least 1.5-year follow-up were reviewed for the current study. All patients signed an informed consent form before a review of their medical records and sending questionnaires. Patients that gave informed consent but did not return the questionnaires were called at least twice. These patients were excluded from the analyses, but their characteristics were included in Online Resource 1. Inclusion criteria were patients with a primary tumor of stages I–III located in the rectosigmoid and rectum treated with surgical resection. Patients who underwent emergency surgery, palliative intended surgery, or who were treated with an APE were excluded. Additionally, patients with < 90% completed questionnaires were excluded. Data regarding 30-day morbidity and mortality were extracted from the Dutch ColoRectal Audit (DCRA), a nationwide clinical audit [24]. The remaining data were extracted from the electronic patient record.

### Baseline characteristics and outcomes

Distance from anus was measured during colonoscopy. Short-term endpoints were 90-day major complications, readmissions, and reinterventions. Major complications were defined according to the Clavien-Dindo classification as ≥ IIIa [25]. The HRQoL of patients was assessed as the primary outcome. Secondary outcomes at 1 and 2 years after surgery were unplanned re-admissions and re-interventions after the initial 30-day postoperative period.

### Health-related quality of life assessment

After at least 1.5 years of follow-up, patients were asked to fill in the HRQoL questionnaires (EORTC QLQ-CR29, EORTC QLQ-C30, and EQ-5D-5L) [26–28]. In all questionnaires, a 4-point Likert scale was used, and subsequently, all responses were linearly converted to 0–100 scales.

### Statistical analyses

The statistical analysis was performed with SPSS Statistics version 24. Patients were divided into two groups, patients who had a stoma at the time of follow-up and patients without a stoma at the time of follow-up. Chi-square test was used for categorical variables; the Mann–Whitney *U* test was used for numeric variables. Multivariate analysis using the linear regression was performed to correct for possible confounding with correction for Charlson comorbidity index and tumor recurrence. For sub-analysis, the population was divided into a group with major LARS and a group of patients with a stoma. After using the EQ-5D-5L questionnaire, a crosstab was made. The *p*-value of the VAS score was calculated using the Mann–Whitney *U* test. The *p*-values of mobility, self-care, usual activity, pain, and anxiety were calculated with Pearson’s chi squared test. A *p*-value of 0.05 was considered statistically significant. In line with current evidence, a HRQoL score difference of > 5% was considered clinically significant [29]. Outcomes were assumed significant if both statistically and clinically significant.

## Results

### Patient characteristics

**Fig. 1:**
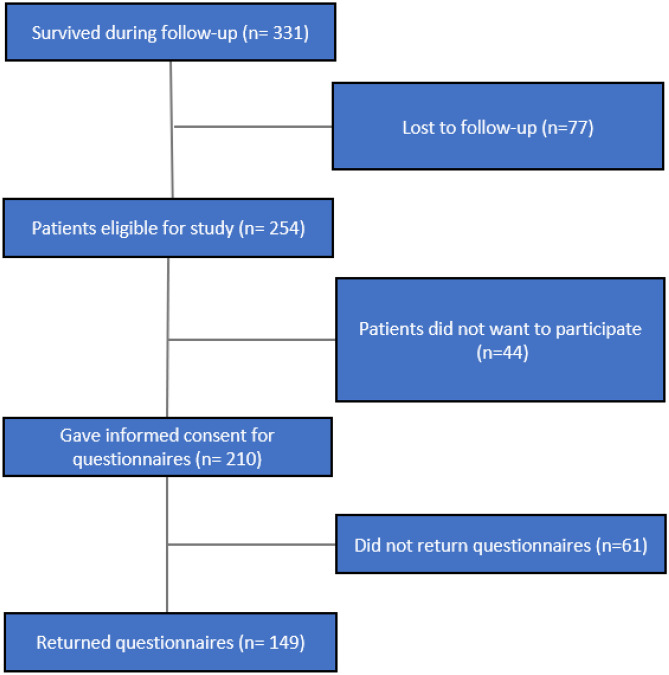
Flowchart patient inclusion

A total of 254 patients were eligible for the study, of which 44 (17.3%) refused to participate. Of the 210 patients that provided informed consent 149 (70.9%) filled out the questionnaires after a mean follow-up of 3.69 (range: 1–8) years (Fig. 1). The 61 patients (29.1%) that did consent to take part in the study but did not return the questionnaires were on average older in both the stoma and no stoma groups; other patient characteristics were comparable with those of patients that have returned the questionnaires (Online Resource 1). At the time of follow-up, 23 included patients (15.4%) had a stoma, of which 20 were a colostoma. In total, 103 (69.1%) patients underwent a low anterior resection (LAR) with primary anastomosis, 30 (20.1%) a LAR with a defunctioning stoma, and 16 (10.7%) a Hartmann resection (Table 1). In 46 patients (30.9%), a stoma was constructed during primary surgery and 9 (6.0%) in patients during a reintervention. Thirty-two patients (21.4%) had a temporary stoma, of which 2 were closed more than a year after surgery. Patients who still had a stoma at the time of follow-up were older (*p* = 0.03), had a lower tumor (*p* =  < 0.01), received more frequent neoadjuvant therapy (*p* = 0.03), and had more major postoperative complications (*p* = 0.03). Patients with a stoma had significantly more unplanned readmissions in both the first (*p* < 0.01) and the second year of follow-up (*p* = 0.03) (Table 2). Moreover, significantly more unplanned reinterventions were performed in the stoma group in both the first (*p* < 0.01) and second years (*p* < 0.01) of follow-up.

**Table 1:** Patient characteristics. *Major complications are defined as a Clavien-Dindo ≥ IIIa

		**Stoma**		
		**No** ***N* = 126 (84.6%)**	**Yes** ***N* = 23 (15.4%)**	***p*-value**
**Age (years)**	**Mean (range)**	64.6 (40–85)	69.1 (56–81)	**0.03**
**Gender %**	**Male**	84 (66.7%)	12 (52.2%)	0.18
	**Female**	42 (33.3%)	11 (47.8%)	
**BMI**	**Mean**	26.40	26.50	0.45
**ASA**	**I–II**	119 (94.4%)	20 (87.0%)	0.19
	**III–IV**	7 (5.6%)	3 (13.0%)	
**Comorbidity**	**Yes**	76 (60.3%)	13 (56.5%)	0.73
	**No**	50 (39.7%)	10 (43.5%)	
**Charlson comorbidity index**	**2–6** **7–11**	109 (86.5%) 17 (13.5%)	21 (91.3%) 2 (8.7%)	0.53
**Previous abdominal surgery**	**Yes**	31 (24.6%)	8 (34.8%)	0.31
	**No**	95 (75.4%)	15 (65.2%)	
**Tumor location**	**Distal** **Middle 1/3** **Proximal** **Unknown**	12 (9.5%) 33 (26.2%) 80 (63.5%) 1 (0.8%)	5 (21.7%) 14 (60.9%) 4 (17.4%) 0 (0.0%)	** < 0.01**
**Tumor stage**	**I**	14 (11.1%)	1 (4.3%)	0.83
	**II**	28 (22.2%)	5 (21.7%)	
	**III**	82 (65.1%)	17 (73.9%)	
	**IIII** **unknown**	1 (0.8%) 1 (0.8%)	0 (0.0%) 0 (0.0%)	
**Neoadjuvant therapy**	**Radiotherapy**	21 (16.7%)	7 (30.4%)	**0.03**
	**Chemoradiation**	27 (21.4%)	9 (39.2%)	
	**None**	78 (61.9%)	7 (30.4%)	
**Minimal invasive**	**Yes**	122 (96.8%)	22 (95.5%)	0.77
	**No**	4 (3.2%)	1 (4.3%)	
**Type of initial surgery**	**LAR**	99 (78.6%)	4 (17.4%)	** < 0.01**
	**LAR with diverting stoma**	26 (20.6%)	4 (17.4%)	
	**Hartmann**	1 (0.8%)	15 (65.2%)	
**Stoma formation**	**During primary surgery**	27 (21.4%)	19 (82.6%)	** < 0.01**
	**During reintervention**	5 (4.1%)	4 (17.4%)	
	**No**	94 (74.5%)	0 (0.0%)	
Major complications*	**Yes**	16 (12.7%)	7 (30.4%)	**0.03**
	**No**	110 (87.3%)	16 (69.6%)	
**Follow-up in years**	**Mean (range)**	3.6 (1–7)	4.4 (2–8)	0.06

**Table 2:** One- and 2-year endpoints. Patients were divided by having a stoma at the time of follow-up. Unplanned readmission and unplanned reinterventions did not include stoma reversal-related admissions and/or stoma reversal interventions

		**Stoma**		**p-value**
		**No**	**Yes**	
		***N* = 126**	***N* = 23**	
**1-year endpoints**				
** Unplanned readmission**	**Yes**	18 (14.3%)	9 (39.1%)	** < 0.01**
	**No**	108 (85.7%)	14 (60.9%)	
** Unplanned re-intervention**	**Yes**	6 (4.8%)	7 (30.4%)	** < 0.01**
	**No**	120 (95.2%)	16 (69.6%)	
**2-year endpoints**				
** Unplanned readmission**	**Yes**	24 (19.0%)	10 (43.5%)	** < 0.01**
	**No**	102 (81.0%)	13 (56.5%)	
** Unplanned re-intervention**	**Yes**	9 (7.1%)	9 (39.1%)	** < 0.01**
	**No**	117 (92.9%)	14 (60.9%)	

### Health-related quality of life

**Fig. 2:**
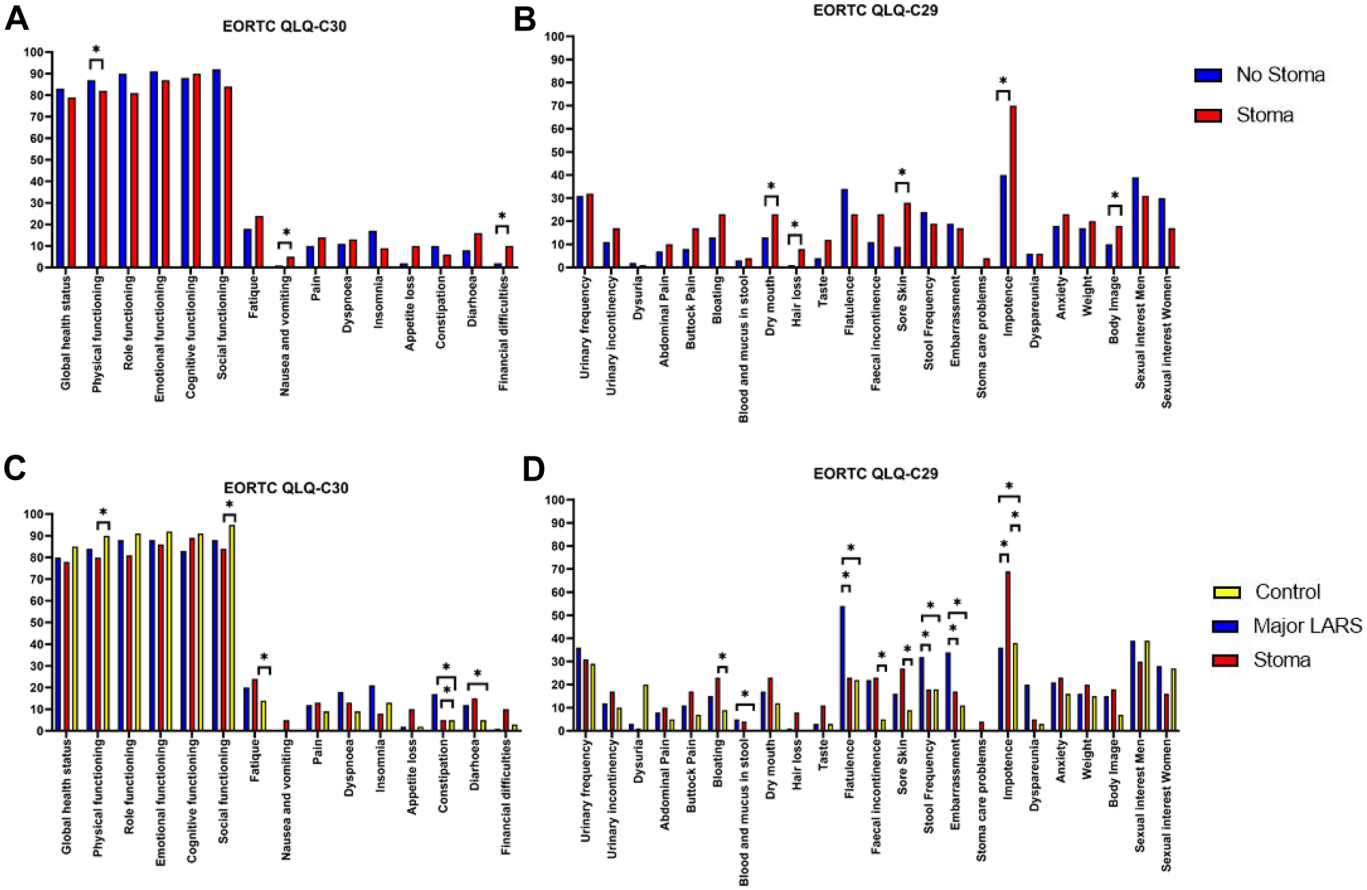
Patient reported outcomes (PROMs). **A** EORTC-QLQ-C30, comparison between patients with stoma (red) and patients without stoma (blue) at follow-up. **B** Patient reported outcomes (PROMs) using EORTC-QLQ-CR29, comparison between patients with stoma and patients without stoma at follow up. **C** EORTC-QLQ-C30**,** comparison between patients with stoma (red), patients major low anterior resection syndrome (LARS) (blue) and control patients, without a stoma or major LARS (yellow) at follow up. **D** EORTC-QLQ-CR29, comparison between patients with stoma and patients major low anterior resection syndrome (LARS) control patients, without a stoma or major LARS (yellow) at follow-up. *p-value < 0.05

The overall quality of life more than 2 years after surgery was not significantly different between patients with and without a stoma, not in the EQ-5D-5L (*p* = 0.28) nor in the EORTC-QLQ-C30 (*p* = 0.15) (Fig. 2; Online Resource 2; Online Resource 4). However, patients with a stoma reported significantly lower physical functioning (*p* = 0.03), significantly more problems with self-care (*p* = 0.03), and usual activity (*p* =  < 0.01). Moreover, patients who received a stoma had significantly more complaints of nausea and vomiting (*p* = 0.02), dry mouth (*p* = 0.03), hair loss (*p* = 0.02), sore skin (*p* < 0.01), impotence (*p* = 0.01), and lower body image (*p* = 0.03) (Fig. 2; Online Resource 3). Additionally, patients with a stoma reported more financial difficulties (*p* = 0.02).

In a multivariate analysis, a stoma present at follow-up was associated with a lower global health status (RR: 0.93, 95%CI 0.88–0.99, *p* = 0.04) and physical functioning (RR: 0.91, 95CI% 0.86–0.96, *p* < 0.01) (Table S5, S6). Also, a higher cT score (RR: 0.97, 95%CI 0.95–0.99, *p* < 0.01) and neoadjuvant chemoradiotherapy (RR: 0.94, 95%CI 0.98–0.99, *p* = 0.02) were associated with a lower global health status (Online Resource 5).

Male sex (RR: 0.95, 95%CI 0.92–0.99, *p* = 0.01), higher ASA score (RR: 0.92, 95%CI 0.89–0.96, *p* < 0.01), a higher cN score (RR: 0.97, 95%CI 0.95–0.99, *p* = 0.01), and Hartmann procedure (RR: 0.90, 95%CI 0.84–0.96, *p* < 0.01) were significantly associated with a lower reported physical functioning (Online Resource 6).

### Major-LARS and health-related quality of life

A sub-analysis was done for patients that did not have a stoma at follow-up and reported major-LARS (*n* = 30, 23.8%). No difference was found in global health status between major LARS patients and patients with a stoma (*p* = 0.50). Furthermore, no significant difference was found for any of the five functioning scales of the EORTC-QLQ-C30 (Fig. 2; Online Resource 7). Within the EORTC-QLQ-CR29, major-LARS patients reported more problems with flatulence (*p* =  < 0.01) and stool frequency (*p* = 0.03) (Fig. 2; Online Resource 8). Moreover, patients with a major-LARS had more complaints of embarrassment compared to patients with a stoma (*p* = 0.02).

## Discussion

This study evaluated the HRQoL in patients with an anastomosis or a stoma 2 years or more after sphincter-sparing rectal resection for cancer. It shows that postoperative global health status and physical functioning are negatively associated with the presence of a stoma in these patients after adjusting for possible cofounders (Charlson comorbidity index, tumor recurrence). In contrast, no clinically significant differences in HRQoL were found between patients with a stoma and patients with an anastomosis and major LARS. Patients with major LARS had more complaints of embarrassment than patients with a stoma. Patients with a stoma had a significantly higher unplanned readmission and reintervention rate in the first 2 years after surgery.

Earlier studies showed ambiguous results for the influence of a stoma on HRQoL. A Cochrane review found that out of the 26 studies included, only 10 reported significantly poorer HRQoL in patients with a permanent stoma [30]. Therefore, the authors concluded their study did not allow for firm conclusions about whether patients with or without permanent colostoma have a superior HRQoL after rectal cancer surgery. One explanation for a reduced quality of life with a stoma can be stoma-related problems. Vonk-Klaasen et al. demonstrated in their systematic review that stoma-related problems, defined as sexual problems, feeling depressed, constipation, body image, difficulties while traveling, and worry about stoma noises, lead to a lower HRQoL [22]. Furthermore, differences in body image were observed, which were most likely caused by the presence of a stoma. In addition, significantly more male patients with a stoma complained about impotence. It should be noted here that the patients with a stoma were significantly older and that some patients were not sexually active anymore at time of surgery. Some of the above reported differences may therefore be at least partly due to the influence of age.

When comparing patients with poor functional outcomes and patients with a stoma, this study did not show differences in HRQoL. Most studies on HRQoL of patients with major LARS only compared patients with and without major-LARS. These studies agree that major LARS is associated with a decreased HRQoL [14, 15, 31, 32]. However, also, patient and treatment characteristics (e.g., age, radiotherapy, low anastomosis) of patients that develop major LARS are likely to influence HRQoL [31, 33]. In this study, patients with major LARS had significantly more complaints of embarrassment than patients with a stoma, which can be an important issue to discuss with a patient when a high risk of major LARS is anticipated. The Pre-Operative LARS score (POLARS) can be used to make an estimation of LARS score to predict the postoperative functional outcome [34].

The current study showed that patients with a stoma had significantly more readmissions and reinterventions. These results are in line with current literature [19, 35]. Additionally, stoma-related complications (e.g., bulge, peristomal hernia) were shown to be associated with a decrease in HRQoL, which could have impacted the results of this study [19, 36]. The increased number of readmissions and reinterventions in patients with a stoma as well as stoma-related complications are also relevant in the tradeoff between a stoma and an anastomosis.

A factor that should be taken into account when comparing different studies on quality of life after rectal cancer surgery is the timing of measuring PROMs [37]. Compared to the population norm, HRQoL improves 3 to 6 months after surgery with patients reaching role-, physical-, and emotional functioning [38, 39]. Studies suggest that HRQoL improvement during this period is caused by fewer defecation or stoma-related complaints, as well as the reversal of temporary stomas, which possibly contributes to this positive effect [39–41]. Furthermore, the age of patients might be an important factor in HRQoL studies after rectal cancer surgery. Recent studies have shown that younger patients (< 65 years) are more affected in their quality of life than elderly patients [38, 39]. Several other studies have shown that the overall quality of life in colorectal cancer survivors is comparable to that of the population norms, suggesting that cancer survivors are very resilient and cope well with their treatment [38, 39, 42]. Colorectal cancer survivors have persisting concerns, such as having to adapt to living with a stoma, these concerns consist of clothing difficulties, dietary changes, and bowel functioning [43]. How well patients cope with these problems hugely influences their quality of life and should be considered regarding PROMs. Additionally, comparison of patients with an anastomosis or a stoma may be troubled by confounding by indication; i.e., the choice for a stoma is influenced by the (perceived) risk of adverse postoperative outcomes. In this study, this is reflected by the fact that patients with a stoma had a more advanced age, lower tumor location, and received more neoadjuvant therapy. In general, advancing age goes hand in hand with a declining HRQoL [44]. This preoperative patient selection and the subsequent difference in patient characteristics and treatment decisions are inevitable in retrospective HRQoL research.

As stated, the decision between an anastomosis and a (temporary) stoma after sphincter-sparing rectal cancer surgery is motivated by the risk of adverse events (e.g., anastomotic leakage) and the expected functional outcomes [12, 33, 45]. However, since this decision is usually not a straightforward one, caused by the lack of a clinically “best choice,” considering the risks of poor functional outcome makes this decision preference-sensitive and therefore particularly relevant for shared decision-making [34, 46, 47]. The presented HRQoL effects of a stoma and major LARS in this study might provide information that can be used as patient information to assist in shared decision-making. Furthermore, explicit patient consideration of the trade-off between anastomosis or a stoma might positively influence the long-term quality of life and lead to a higher acceptance of possible consequences [48].

### Limitations

The fact that this study excluded all patients that underwent an APE could be scrutinized. However, with a classic APE, there is no decision to be made between a stoma or an anastomosis, as the latter is not an option. Furthermore, APE patients typically have lower rectal tumors with invasion of the sphincter complex or sphincter insufficiency, which is associated with typical and worse pre-operative symptoms [35, 49]. Nonetheless, patients could have been excluded that had intersphincteric APEs as an alternative for a Hartmann. In these patients, the same considerations about an anastomosis or a stoma could have been made, but surgeons could have been reluctant to leave the rectal stump. The decision whether to perform an APE as an alternative to a low Hartmann is an ongoing debate; the main reason this is done is to avoid the risk of staple line rupture and subsequent leakage and pelvic abscesses as well as persisting mucus production and diversion proctitis [50, 51]. However, an APE is associated with additional risks of perineal wound complications [52]. In our hospitals, the rectal stump is typically left in place except in very low resections. Another limitation of this study was the small sample size, especially in the stoma group. The latter could have been consequential to the exclusion of APE patients as mentioned above and stoma reversal before follow-up and answering the PROM questionnaires. An additional limitation is that the sample size did not allow for sub-analysis of patients with an ileostoma and a colostoma or stoma formation during primary surgery and stoma formation during reintervention. Furthermore, a limitation is the variation in follow-up. In this study, we included all patients operated from 2012 until 2016. The follow-up and time of receiving the questionnaires after operation varied between 2 and 7 years. However, to our knowledge, this is the first study to make a comparison of long-term HRQoL between patients with a stoma and major LARS.

## Conclusion

This study shows that after correction for possible confounders, a stoma is associated with a lower global health status and physical functioning. However, no clinically significant difference was found in HRQoL between patients with major LARS and patients with a stoma. This suggests that the choice between stoma and anastomosis is mainly preferential and should be made together with the patient. This study offers leads for improved patient information and enhanced shared decision-making before rectal cancer surgery.

## Supplementary Information

Below is the link to the electronic supplementary material.

Supplementary file1 (DOCX 47 KB)
