# Supplementary material for: Stoma versus anastomosis after sphincter-sparing rectal cancer resection; the impact on health-related quality of life
Source: Int J Colorectal Dis. 2022 Sep 26;37(10):2197–205. doi: 10.1007/s00384-022-04257-w (PMC9560940; doi:10.1007/s00384-022-04257-w)
Supplement: Supplementary file 1 — Supplementary file1 (DOCX 47 KB) [file 384_2022_4257_MOESM1_ESM.docx]

**SUPPLEMENTARY INFORMATION – International Journal of Colorectal Disease**

**Stoma Versus Anastomosis After Sphincter-Sparing Rectal Cancer Resection; the Impact on Health-Related Quality of Life**

Jelle P.A. Algie, Bsc.^1^*, Robert T. van Kooten, MD^1^*^#^, Rob A.E.M. Tollenaar MD, PhD^1^, Michel W.J.M. Wouters MD, PhD^2,3^, Koen C.M.J. Peeters MD, PhD^1^, Jan Willem T. Dekker MD, PhD^4^

^1^ Department of Surgery, Leiden University Medical Center, Leiden, The Netherlands

^2^ Department of Biomedical Data Sciences, Leiden University Medical Center, Leiden, The Netherlands

^3^ Department of Surgery, Netherlands Cancer Institute-Antoni van Leeuwenhoek, Amsterdam, The Netherlands

^4^ Department of Surgery, Reinier de Graaf Gasthuis, Delft, The Netherlands

**Online Resource 1 -** Patient characteristics of non-responders

* Major complications defined as Clavien-Dindo ≥ IIIa.

|  |  | **Stoma** |  |
| --- | --- | --- | --- |
|  |  | **No**  **N=53 (86.9%)** | **Yes**  **N=8 (13.1%)** |
| **Age (years)** | **Mean (range)** | 68.3 (37-85) | 75.4 (63-84) |
| **Gender %** | **Male** | 31 (58.5%) | 4 (50%) |
|  | **Female** | 22 (41.5%) | 4 (50%) |
| **BMI** | **Mean** | 25.8 | 25.6 |
| **ASA** | **I-II** | 50 (94.3%) | 6 (75.0%) |
|  | **III-IV** | 3 (5.7%) | 2 (25.0%) |
| **Comorbidity** | **Yes** | 35 (66.0%) | 7 (85.5%) |
|  | **No** | 18 (34.0%) | 1 (12.5%) |
| **Charlson Comorbidity index** | **2-6**  **7-11** | 47 (88.7%)  6 (11.3%) | 5 (62.5%)  3 (37.5%) |
| **Previous abdominal**  **surgery** | **Yes** | 16 (30.2%) | 2 (25.0%) |
|  | **No** | 37 (69.8%) | 6 (75.0%) |
| **Tumor location** | **Distal**  **Middle 1/3**  **Proximal**  **Unknown** | 5 (9.4%)  22 (41.5%)  26 (49.1%)  0 (0.0%) | 2 (25.0%)  6 (75.0%)  0 (0.0%)  0 (0.0%) |
| **Tumor cStage** | **I** | 3 (5.7%) | 0 (0.0%) |
|  | **II** | 17 (32.1%) | 3 (37.5%) |
|  | **III** | 32 (60.4%) | 5 (62.5%) |
|  | **IIII**  **unknown** | 1 (1.9%)  0 (0.0%) | 0 (0.0%)  0 (0.0%) |
| **Neoadjuvant**  **therapy** | **Radiotherapy** | 12 (22.6%) | 3 (37.5%) |
|  | **Chemoradiation** | 8 (15.1%) | 1 (12.5%) |
|  | **None** | 33 (62.3%) | 4 (50.0%) |
| **Minimal invasive** | **Yes** | 48 (90.6%) | 6 (75.0%) |
|  | **No** | 5 (9.4%) | 2 (25.0%) |
| **Type of initial surgery** | **LAR** | 35 (66.0%) | 0 (0.0%) |
|  | **LAR with diverting stoma** | 18 (34.0%) | 3 (37.5%) |
|  | **Hartmann** | 0 (0.0%) | 5 (62.5%) |
| **Major complications*** | **Yes** | 10 (18.9%) | 1 (12.5%) |
|  | **No** | 43 (81.1%) | 7 (87.5%) |
| **Follow-up in years** | **Mean (range)** | 3.8 (1-6) | 4.0 (1-6) |

**Online Resource 2 -** Patient Reported Outcomes (PROMs) using EORTC-QLQ-C30, comparison between patients with stoma and without a stoma. *A value of 0 is considered as a low quality of life, a value of 100 is considered as a maximum quality of life. ^#^A value of 0 is considered as a low level of complication, a value of 100 is considered as a maximum level of complication of functioning

|  | **Stoma** | |  |
| --- | --- | --- | --- |
|  | **No**  **N=126** | **Yes**  **N=23** | **p-value** |
| **Global Health status *** | 82.87 | 78.62 | 0.15 |
| **Physical functioning *** | 88.63 | 80.87 | **0.03** |
| **Role Functioning *** | 89.52 | 81.16 | 0.09 |
| **Emotional functioning *** | 90.79 | 86.60 | 0.41 |
| **Cognitive functioning *** | 88.27 | 89.85 | 0.97 |
| **Social functioning *** | 92.40 | 84.06 | 0.06 |
| **Fatigue**  ^#^ | 17.95 | 24.15 | 0.11 |
| **Nausea and vomiting** ^#^ | 0.79 | 5.07 | **0.02** |
| **Pain**  ^#^ | 9.60 | 13.77 | 0.27 |
| **Dyspnoea**  ^#^ | 11.20 | 13.04 | 0.82 |
| **Insomnia**  ^#^ | 16.67 | 8.69 | 0.25 |
| **Appetite loss**  ^#^ | 2.11 | 10.14 | 0.15 |
| **Constipation**  ^#^ | 9.60 | 5.79 | 0.32 |
| **Diarrhoea**  ^#^ | 8.33 | 15.94 | 0.24 |
| **Financial difficulties**  ^#^ | 1.60 | 10.14 | **0.02** |

**Online Resource 3** - Patient Reported Outcomes (PROMs) using EORTC-QLQ-CR29. A value of 0 is considered as a low level of a complication, a value of 100 is considered as a maximum level.

|  |  | **Stoma** | |  |
| --- | --- | --- | --- | --- |
|  |  | **No**  **N=126** | **Yes**  **N=23** | **p-value** |
| **Urinary frequency** | | 31.32 | 31.88 | 0.77 |
| **Urinary incontinency** | | 11.11 | 17.39 | 0.21 |
| **Dysuria** | | 2.17 | 1.45 | 0.79 |
| **Abdominal Pain** | | 6.99 | 10.15 | 0.87 |
| **Buttock Pain** | | 8.06 | 17.39 | 0.07 |
| **Bloating** | | 12.63 | 23.19 | 0.06 |
| **Blood and mucus in stool** | | 3.63 | 4.35 | 0.59 |
| **Dry mouth** | | 13.44 | 23.19 | **0.03** |
| **Hair loss** | | 0.81 | 8.70 | **0.02** |
| **Taste** | | 3.79 | 11.59 | 0.06 |
| **Flatulence (without stoma)** | | 33.88 |  | - |
| **Faecal incontinence (without stoma)** | | 11.29 |  | - |
| **Sore Skin (without stoma)** | | 9.92 |  | **-** |
| **Stool Frequency (without stoma)** | | 23.83 |  | - |
| **Embarrassment (without stoma)** | | 19.01 |  | - |
| **Flatulence (with stoma)** | |  | 23.19 | - |
| **Faecal incontinence/leakage (with stoma)** | |  | 23.19 | - |
| **Sore Skin (with stoma)** | |  | 27.54 | - |
| **Stool Frequency/bags change (with stoma)** | |  | 18.84 | - |
| **Embarrassment (with stoma)** | |  | 17.39 | - |
| **Stoma care problems** | | - | 4.35 | - |
| **Impotence** | | 39.99 | 69.70 | **0.01** |
| **Dyspareunia** | | 6.45 | 5.56 | 1.00 |
| **Anxiety** | | 17.60 | 23.19 | 0.37 |
| **Weight** | | 16.53 | 20.29 | 0.90 |
| **Body Image** | | 9.99 | 18.36 | **0.03** |
| **Sexual interest Men** | | 38.89 | 30.55 | 0.33 |
| **Sexual interest Women** | | 29.72 | 16.67 | 0.16 |

|  |  | **Stoma** | | |  |  |
| --- | --- | --- | --- | --- | --- | --- |
|  |  | **No**  **N=123** | **Yes**  **N=22** | | **Total**  **N=145** | **p-value** |
| **Mobility** | **1** | 90 (73.2%) | 13 (59.1%) | | 103 (71.0%) | 0.35 |
|  | **2** | 22 (17.9%) | 7 (31.8%) | | 29 (20.0%) |  |
|  | **3** | 9 (7.3%) | 1 (4.5%) | | 10 (6.9%) |  |
|  | **4** | 2 (1.6%) | 1 (4.5%) | | 3 (2.1%) |  |
|  | **5** | 0 (0.0%) | 0 (0.0%) | | 0 (0.0%) |  |
| **Self-care** | **1** | 118 (95.9%) | 18 (81.8%) | | 136 (93.8%) | **0.03** |
|  | **2** | 3 (2.4%) | 3 (13.6%) | | 6 (4.1%) |  |
|  | **3** | 2 (1.7%) | 1 (4.5%) | | 3 (2.1%) |  |
|  | **4** | 0 (0.0%) | 0 (0.0%) | | 0 (0.0%) |  |
|  | **5** | 0 (0.0%) | 0 (0.0%) | | 0 (0.0%) |  |
| **Usual activity** | **1** | 98 (79.7%) | 10 (45.5%) | | 108 (74.5%) | **<0.01** |
|  | **2** | 15 (12.2%) | 6 (27.3%) | | 21 (14.5%) |  |
|  | **3** | 8 (6.5%) | 5 (22.7%) | | 13 (9.0%) |  |
|  | **4** | 2 (1.6%) | 1 (4.5%) | | 3 (2.1%) |  |
|  | **5** | 0 (0.0%) | 0 (0.0%) | | 0 (0.0%) |  |
| **Pain or discomfort** | **1** | 72 (58.5%) | 13 (59.1%) | | 85 (58.6%) | 0.36 |
|  | **2** | 37 (30.1%) | 9 (40.9%) | | 46 (31.7%) |  |
|  | **3** | 11 (9.0%) | 0 (0.0%) | | 11 (7.6%) |  |
|  | **4** | 3 (2.4%) | 0 (0.0%) | | 3 (2.1%) |  |
|  | **5** | 0 (0.0%) | 0 (0.0%) | | 0 (0.0%) |  |
| **Anxiety or Depression** | **1** | 104 (84.6%) | 15 (68.2%) | | 119 (82.1%) | 0.13 |
|  | **2** | 14 (11.3%) | | 6 (27.3%) | 20 (13.8%) |  |
|  | **3** | 5 (4.1%) | 1 (4.5%) | | 6 (4.1%) |  |
|  | **4** | 0 (0.0%) | 0 (0.0%) | | 0 (0.0%) |  |
|  | **5** | 0 (0.0%) | 0 (0.0%) | | 0 (0.0%) |  |
| **VAS** | **mean** | 82.85 | 76.41 | |  | 0.28 |

**Online Resource 4 -** Patient Reported Outcomes (PROMs) using EQ-5D-5L, Patient Reported Outcomes (PROMs) using EORTC-QLQ-C30, comparison between patients with stoma and without a stoma. A value of 1 is considered as no problems, a value of 2 as slight problems, a value of 3 as moderate problems, a value of 4 as severe problems and a value of 5 as unable to.

**Online Resource 5** **–** Individual factors influencing the global health status, measured by the EORTC-QLQ-C30.

*Corrected for: Charlson comorbidity index, tumor recurrence. ^#^Major complications defined as Clavien-Dindo ≥ IIIa.

| **Global Health Status** | **Univariate** | **p-value** | **Multivariate*** | **p-value** |
| --- | --- | --- | --- | --- |
| Age | 1.00 (1.00-1.00) | 0.23 | - |  |
| Male sex | 0.98 (0.95-1.02) | 0.36 | 0.98 (0.95-1.02) | 0.33 |
| ASA-score | 0.98 (0.95-1.02) | 0.33 | 1.00 (0.96-1.03) | 0.81 |
| Charlson Comorbidity index | 0.98 (0.95-0.99) | **<0.01** | - |  |
| Stoma at follow-up | 0.95 (0.90-1.00) | **0.04** | 0.93 (0.88-0.99) | **0.01** |
| Comorbidity | 1.01 (0.97-1.04) | 0.66 | - |  |
| Major complications^#^ | 0.99 (0.92-1.02) | 0.69 | 0.98 (0.91-1.01) | 0.10 |
| Tumor recurrence | 0.86 (0.80-0.93) | **<0.01** | - |  |
| Neoadjuvant chemoradiotherapy | 0.96 (0.91-1.01) | 0.12 | 0.94 (0.89-0.99) | **0.02** |
| Adjuvante chemotherapy | 1.01 (0.98-1.04) | 0.46 | 1.02 (0.96-1.08) | 0.56 |
| cT-score | 0.94 (0.92-0.96) | **<0.01** | 0.97 (0.95-0.99) | **<0.01** |
| cN-score | 0.98 (0.96-1.01) | 0.16 | 0.98 (0.96-1.00) | 0.11 |

**Online Resource 6 –** Individual factors influencing the physical functioning, measured by the EORTC-QLQ-C30.

*Corrected for: Charlson comorbidity index, tumor recurrence. ^#^Major complications defined as Clavien-Dindo ≥ IIIa

| **Physical Functioning** | **Univariate** | **p-value** | **Multivariate*** | **p-value** |
| --- | --- | --- | --- | --- |
| Age | 0.99 (0.99-1.00) | **<0.01** | - |  |
| Male sex | 0.96 (0.92-0.99) | **0.02** | 0.95 (0.92-0.99) | **0.01** |
| ASA-score | 0.91 (0.88-0.94) | **<0.01** | 0.92 (0.89-0.96) | **<0.01** |
| Charlson Comorbidity index | 0.97 (0.96-0.99) | **<0.01** | - |  |
| Stoma at follow-up | 0.91 (0.86-0.96) | **<0.01** | 0.91 (0.86-0.96) | **<0.01** |
| Comorbidity | 0.94 (0.91-1.98) | **<0.01** | - |  |
| Major complications^#^ | 1.00 (0.96-1.04) | 0.68 | 0.99 (0.95-1.04) | 0.81 |
| Tumor recurrence | 0.98 (0.93-1.02) | 0.6 | - |  |
| Neoadjuvant chemoradiotherapy | 0.98 (0.93-1.03) | 0.45 | 0.97 (0.92-1.02) | 0.17 |
| Adjuvant chemotherapy | 1.01 (0.98-1.04) | 0.6 | 1.01 (0.95-1.07) | 0.77 |
| cT-score | 0.99 (0.97-1.01) | **0.19** | 0.98 (0.96-1.01) | 0.13 |
| cN-score | 0.97 (0.95-0.99) | **0.02** | 0.97 (0.95-0.99) | **0.01** |

**Online Resource 7 -** Patient Reported Outcomes (PROMs) using EORTC-QLQ-CR30, comparison between patients with stoma and with major Low Anterior Resection Syndrome (LARS). *A value of 0 is considered as a low quality of life, a value of 100 is considered as a maximum quality of life. ^#^A value of 0 is considered as a low level of complication, a value of 100 is considered as a maximum level of complication.

α: statistically different from Major Lars

β: statistically different from Stoma

|  | **Control**  **N=96** | **Major Lars N=30** | **Stoma**  **N=23** | **p-value** |
| --- | --- | --- | --- | --- |
| **Global Health status *** | 84.81 | 80.83 | 78.62 | 0.39 |
| **Physical functioning *** | 89.75^β^ | 84.26 | 80.87 | 0.13 |
| **Role Functioning *** | 90.72 | 88.89 | 81.16 | 0.41 |
| **Emotional functioning *** | 92.20 | 88.89 | 86.60 | 0.49 |
| **Cognitive functioning *** | 90.72 | 83.33 | 89.85 | 0.09 |
| **Social functioning *** | 94.51 ^β^ | 88.33 | 84.06 | **0.02** |
| **Fatigue**  ^#^ | 14.34 ^β^ | 20.00 | 24.15 | 0.32 |
| **Nausea and vomiting** ^#^ | 0.21 | 0.56 | 5.07 | **<0.01** |
| **Pain**  ^#^ | 8.86 | 12.22 | 13.77 | 0.96 |
| **Dyspnoea**  ^#^ | 8.75 | 18.39 | 13.04 | 0.14 |
| **Insomnia**  ^#^ | 12.91 | 21.11 | 8.69 | 0.42 |
| **Appetite loss**  ^#^ | 2.08 | 2.22 | 10.14 | 0.14 |
| **Constipation**  ^#^ | 5.00 ^α^ | 17.24^β^ | 5.79 | **0.01** |
| **Diarrhoea**  ^#^ | 5.49 ^α^ | 12.64 | 15.94 | 0.01 |
| **Financial difficulties**  ^#^ | 2.53 | 1.11 | 10.14 | **<0.01** |

**Online Resource 8** **-** Patient Reported Outcomes (PROMs) using EORTC-QLQ-C29, comparison between patients with stoma and with major Low Anterior Resection Syndrome (LARS). A value of 0 is considered as a low level of a complication, a value of 100 is considered as a maximum level.

α: statistically different from Major Lars

β: statistically different from Stoma

|  | **Control**  **N=96** | **Major LARS N=30** | **Stoma**  **N=23** | **p-value** |
| --- | --- | --- | --- | --- |
| **Urinary frequency** | 29.37 | 36.11 | 31.88 | 0.24 |
| **Urinary incontinency** | 10.42 | 12.64 | 17.39 | 0.41 |
| **Dysuria** | 20.81 | 3.45 | 1.45 | 0.43 |
| **Abdominal Pain** | 5.42 | 8.89 | 10.15 | 0.41 |
| **Buttock Pain** | 7.08 | 11.11 | 17.39 | 0.10 |
| **Bloating** | 9.17 ^β^ | 15.55 | 23.19 | 0.12 |
| **Blood and mucus in stool** | 0.83 ^α^ | 5.00 | 4.35 | **<0.01** |
| **Dry mouth** | 12.50 | 17.78 | 23.19 | 0.58 |
| **Hair loss** | 0.83 | 1.11 | 8.70 | 0.92 |
| **Taste** | 3.33 | 3.45 | 11.59 | 0.54 |
| **Flatulence (without stoma)** | 22.81 ^α^ | 54.02 | - | **<0.01** |
| **Faecal incontinence (without stoma)** | 5.26 ^α,^ | 22.99 | - | **<0.01** |
| **Sore Skin (without stoma)** | 9.21 | 16.09 | - | 0.19 |
| **Stool Frequency (without stoma)** | 18.86 ^α^ | 32.18 | - | **0.01** |
| **Embarrassment (without stoma)** | 11.40 ^α^ | 34.48 | - | **<0.01** |
| **Flatulence (with stoma)** |  |  | 23.19 | **-** |
| **Faecal incontinence/leakage (with stoma)** |  |  | 23.19 | **-** |
| **Sore Skin (with stoma)** |  |  | 27.54 | **-** |
| **Stool Frequency/bags change (with stoma)** |  |  | 18.84 | **-** |
| **Embarrassment (with stoma)** |  |  | 17.39 | **-** |
| **Stoma care problems** | - | - | 4.35 | - |
| **Impotence** | 38.89 ^α β^ | 36.23^β^ | 69.70 | **0.69** |
| **Dyspareunia** | 3.17 | 20.00 | 5.56 | 0.08 |
| **Anxiety** | 16.25 | 21.11 | 23.19 | 0.43 |
| **Weight** | 15.83 | 16.67 | 20.29 | 0.79 |
| **Body Image** | 7.99 ^β^ | 15.55 | 18.36 | **<0.01** |
| **Sexual interest Men** | 39.10 | 39.13 | 30.55 | 0.94 |
| **Sexual interest Women** | 27.54 | 28.57 | 16.67 | 0.45 |
